# Creation of a replicable anatomic model of terrible triad of the elbow

**DOI:** 10.1186/s13018-024-05069-0

**Published:** 2024-10-08

**Authors:** Antoine Baltassat, Florent Baldairon, Samuel Berthe, Alexandre Bellier, Nadia Bahlouli, Philippe Clavert

**Affiliations:** 1 Service de Chirurgie du Membre Supérieur, Hôpital de Hautepierre 2 - CHU Strasbourg, Avenue Molière, Strasbourg, 67000 France; 2 ICube laboratory, University of Strasbourg/CNRS, 2 rue Boussingault, Strasbourg, 67000 France; 3 Univ. Grenoble Alpes, LADAF, CIC INSERM 1406, AGEIS, Grenoble, France

**Keywords:** Elbow dislocation, Elbow fracture, Instability, Anatomic model, Terrible triad, Elbow biomechanics

## Abstract

**Background:**

Terrible triad of the elbow (TTE) is a complex dislocation associating radial head (RH) and coronoid process (CP) fractures. There is at present no reproducible anatomic model for TTE, and pathophysiology is unclear. The main aim of the present study was to create and validate an anatomic model of TTE. Secondary objectives were to assess breaking forces and relative forearm rotation with respect to the humerus before dislocation.

**Methods:**

An experimental comparative study was conducted on 5 fresh human specimens aged 87.4 ± 8.6 years, testing 10 upper limbs. After dissection conserving the medial and lateral ligaments, interosseous membrane and joint capsule, elbows were reproducibly positioned in maximal pronation and 15° flexion, for axial compression on a rapid (100 mm/min) or slow (10 mm/min) protocol, applied by randomization between the two elbows of a given cadaver, measuring breaking forces and relative forearm rotation with respect to the humerus before dislocation.

**Results:**

The rapid protocol reproduced 4 posterolateral and 1 divergent anteroposterior TTE, and the slow protocol 5 posterolateral TTE. Mean breaking forces were 3,126 ± 1,066 N for the lateral collateral ligament (LCL), 3,026 ± 1,308 N for the RH and 2,613 ± 1,120 N for the CP. Comparing mean breaking forces for all injured structures in a given elbow on the rapid protocol found a p-value of 0.033. Comparison of difference in breaking forces in the three structures (LCL, RH and CP) between the slow and rapid protocols found a mean difference of -4%. Mean relative forearm rotation with respect to the humerus before dislocation was 1.6 ± 1.2° in external rotation.

**Conclusions:**

We create and validate an anatomic model of TTE by exerting axial compression on an elbow in 15° flexion and maximal pronation at speeds of 100 and 10 mm/min.

## Background

The elbow joint is a complex structure with significant functional importance being the second most frequently dislocated joint. Dislocation typically occur in a posterolateral direction [1] and are classified as simple when there are capsule-ligament lesion without bone lesion [2], or complex when bone damage is present.

Terrible triad of the elbow (TTE) involves a complex elbow dislocation with radial head (RH) and coronoid process (CP) fractures. Named by Hotchkiss, it’s characterized by its challenging management [3] due to lesions in, at least, two primary elbow stabilizers and three secondary stabilizers, as described by O’Driscoll & al. [4], often leading to chronic instability”.

While studies have explored the pathophysiology of TTE, including lesion mechanisms and chronology [5–11], they’ve been limited and haven’t aimed to develop a reproducible anatomical model [7, 12–15]. Important biomechanical aspects, such as loads to failure and relative forearm rotation with respect to the humerus prior to dislocation, remain poorly understood.

The primary objective of the present study was to create and validate an anatomic model of TTE. The secondary objectives were to assess and compare load to failure and relative forearm rotation with respect to the humerus prior to dislocation.

The study hypothesis was that it would be possible to create and validate an anatomic model of TTE while applying an axial compression in an elbow in sub flexion and pronation.

## Methods

It was a comparative experimental study conducted on 5 fresh human cadavers, with a mean age of 87.4 ± 8.6 years, comprising 3 females, 2 males; resulting in 10 elbows being tested.

All elbows had no prior medical or surgical history, inspection and X-rays were used to confirm the absence of bone deformities, malformations, malignancy, or foreign bodies. Range of motion tests were performed to ensure there was no stiffness.

Bone sections were consistently made 80 mm from the epicondyles on the humerus and 150 mm from the olecranon on the forearm, allowing for the epicondyles to be positioned adjacent to the test bench center of rotation with 3-dimensional displacement under compression.

A single surgeon prepared all elbows, removing all soft tissue except for the medial and lateral collateral ligaments, interosseous membrane, joint capsule, annular ligament, and tissue 2 mm distal to the brachial muscle insertion, to avoid injuring the capsule and CP. Soft tissues were elevated from the humeral, radial and ulnar diaphysis using a periosteal elevator.

TTE is typically caused by a fall onto a hand in extension [8]. Several studies reproduced TTE by axial compression [7, 12, 15]. We used the Instron Electropuls E10000^®^ dynamic test device, with 10 kN electromechanical linear by a bi-axial load cell.

Previous anatomical studies reproduced TTE in full extension [7] and 0–30° flexion [12, 15]. We positioned the elbows in 15° flexion.

Fitzpatrick & al. produced TTE by axial compression in a forearm in pronation [7]. We positioned the elbows in maximal pronation.

TTE classically implicates valgus stress [8, 10, 16, 17], but some studies reproduced it under varus stress [7, 15]. Given this lack of consensus and the intra- and inter-individual variations in physiological valgus according to numerous factors such as handedness, age, gender, ethnicity, etc [18, 19]. , we left the elbows in their native position without extra varus or valgus forces.

**Fig. 1:**
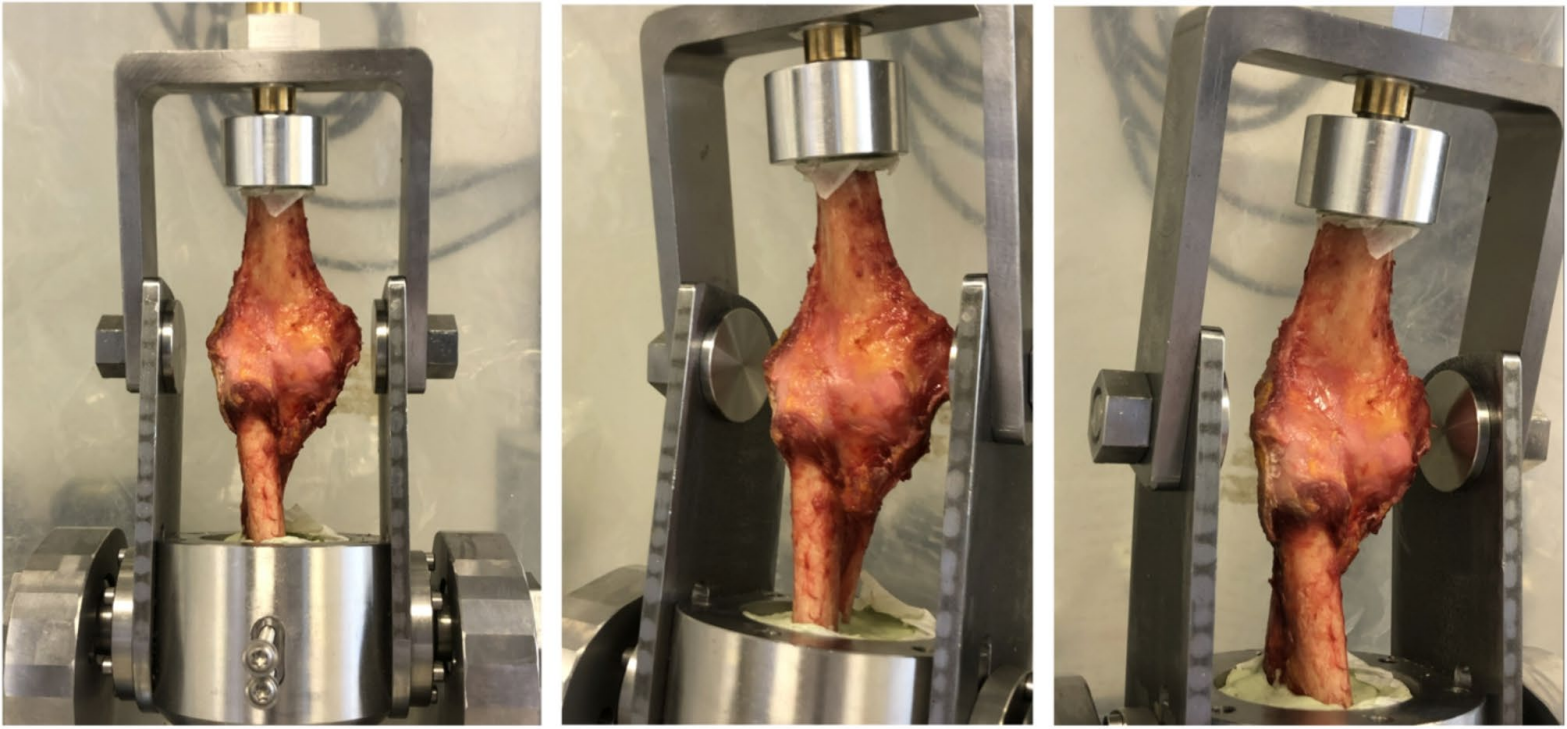
Specimen on test bench (respectively: anterior, anteromedial, anterolateral sides)

The test bench was inclined at 15°, with the superior part vertical. The elbow was positioned with the epicondyles adjacent to the axis of rotation of the device and thus aligned in the frontal plane, with the humerus vertical in the axis of the device. The humeral shaft was fixed using Palacos^®^ surgical cement. The ulnar and radial shafts were cemented in maximal pronation. The metal interfaces of the proximal and distal fixations were solidly attached to the compression device (Fig. 1).

To the best of our knowledge, two studies successfully modeled TTE, but without focusing on reproducibility [7, 15]. We applied two different protocols, rapid and slow, randomized between the two elbows of a each cadaver using computer-generating randomization.

### Rapid protocol

The rapid protocol aimed to mimic the in-vivo lesion mechanism, as dislocation is a sudden event [20]. The displacement rate was 100 mm/min, following Fitzpatrick & al. [7].

Loads to failure were evaluated, but due to the nature of this protocol, stereo-correlation assessment of relative forearm rotation with respect to the humerus prior to dislocation was not feasible.

### Slow protocol

The slow protocol also replicated the traumatic context of TTE and measured forces, but additionally allowed for the measurement of relative forearm rotation with respect to the humerus prior to dislocation. The displacement rate was 10 mm/min, following Wake & al. [15] relative forearm rotation with respect to the humerus prior to dislocation was determined using digital images correlation methods. This non-contact imaging technique measured displacement and rotation of the entire imaged area and involved creating a pattern on the bone surface. A unique pattern of random speckles was applied on the specimens 10 min prior to axial compression, to avoid affecting tissue biomechanics through dehydration. The speckle pattern was artificially created by spraying black paint on the white background of the specimen surface. Subsequently, using two pre-calibrated video cameras operating at a speed of 10 Hz (Hz), relative forearm rotation with respect to the humerus prior to dislocation was quantified in degrees by GOM Correlate^®^ software based on the last image prior to dislocation.

### Points in common between the 2 protocols

A pre-loading of 10 Newtons (N) was applied to all models.

Loading, measured in Newtons, and cylinder displacement, measured in millimeters (mm), were recorded at an acquisition frequency of 10,000 Hz, to determine load to failure in each structure, corresponding to the moment when the linear ascending phase of the load-displacement curve exhibited an inflection point.

Valgus or varus elbow displacement under axial compression was also quantified.

Compression was was stopped when the load exceeded 6,500 N, when there was a sudden 80% drop in maximal force, or when displacement exceeded 50 mm; these thresholds were well above the mean 2,355 N load to failure for TTE reported by Fitzpatrick & al. [7], and we expected a curve comprising several plateaus with a first sudden drop in force associated with the capsule-ligament rupture preceding bone fracture [7, 11] and did not wish to stop compression between the two.

Dislocation occurrence and direction (posterolateral, posteromedial, anterior or divergent) were determined from the position of the ulna and radius relative to the humerus on the test bench.

Afterward, the model was then removed from the device, and dissection examined any RH or CP fractures, which were classified according to Mason and Regan-Morrey.

### Statistics

Qualitative variables were presented as counts and percentage while quantitative variables were reported as means and standard deviations.

Univariate analysis utilized non-parametric Kruskal-Wallis tests. In case of significance at the 5% threshold, pairwise comparison was conducted, using the Dwass-Steel-Critchlow-Fligner tests. Analyses were performed using Jamovi software, version 2.3 (The Jamovi Project, Sydney, Australia).

## Results

### Reproducibility of the model

The rapid protocol reproduced posterolateral TTE in 4 out of 5 cases (80%) and divergent anteroposterior TEE in 1 out of 5 (20%). The slow protocol reproduced posterolateral TTE in 5 out of 5 cases (100%).

**Fig. 2:**
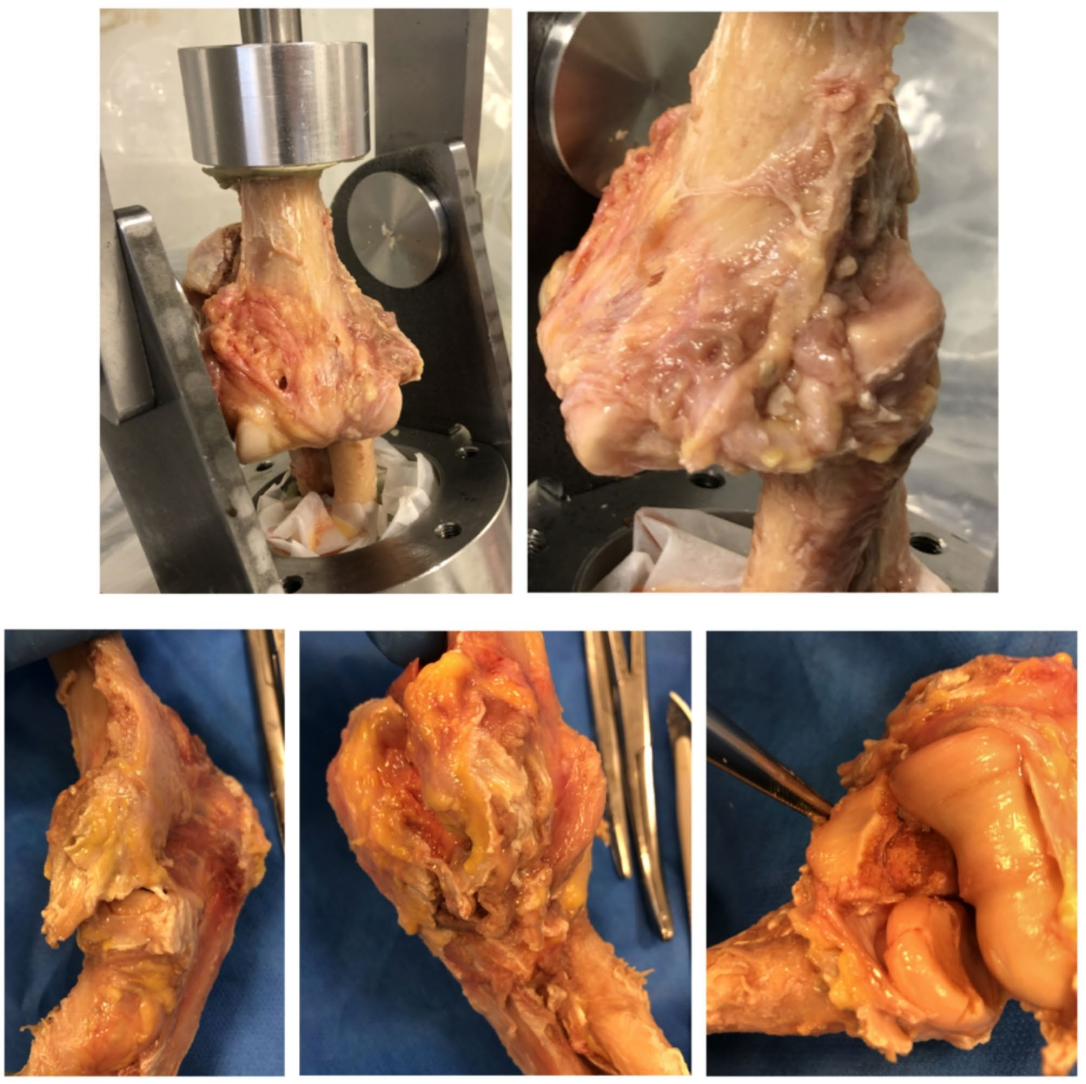
Posterolateral TTE lesions (respectively: anteromedial and anterolateral sides with posterolateral luxation, lateral side with LCL tear, medial side without MCL lesion, anterior side with TR and PC fractures)

TTE with posterolateral dislocation systematically involved RH and CP fractures, LCL tear and anterior and posterior capsule rupture, without medial collateral ligament (MCL) lesions (Fig. 2).

All the RH fractures involved the head only, no fracture of the neck of the radius was observed.

**Fig. 3:**
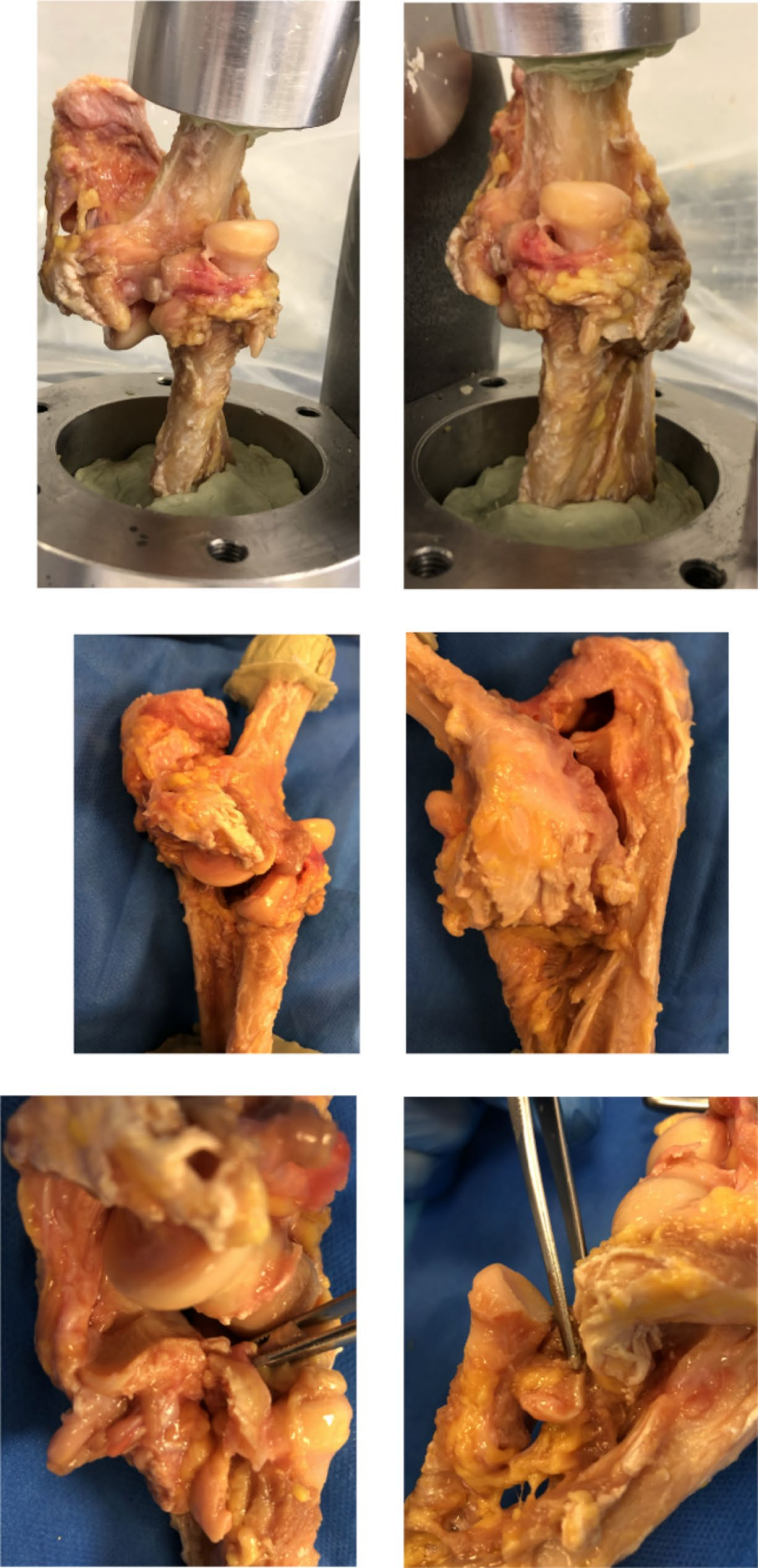
Divergent anteroposterior TTE lesions. *Legend*: Respectively: anteromedial and anterolateral sides with divergent anteroposterior luxation, medial and lateral sides with LCL and MCL tears and annular ligament and interosseous membrane ruptures, medial and lateral sides with PC and TR fractures

TTE with divergent anteroposterior dislocation involved RH and CP fractures, LCL and MCL tears, anterior and posterior capsule rupture, annular ligament tear and interosseous membrane rupture (Fig. 3).

Table 1 details lesion assessment in each TTE. 

**Table 1:**
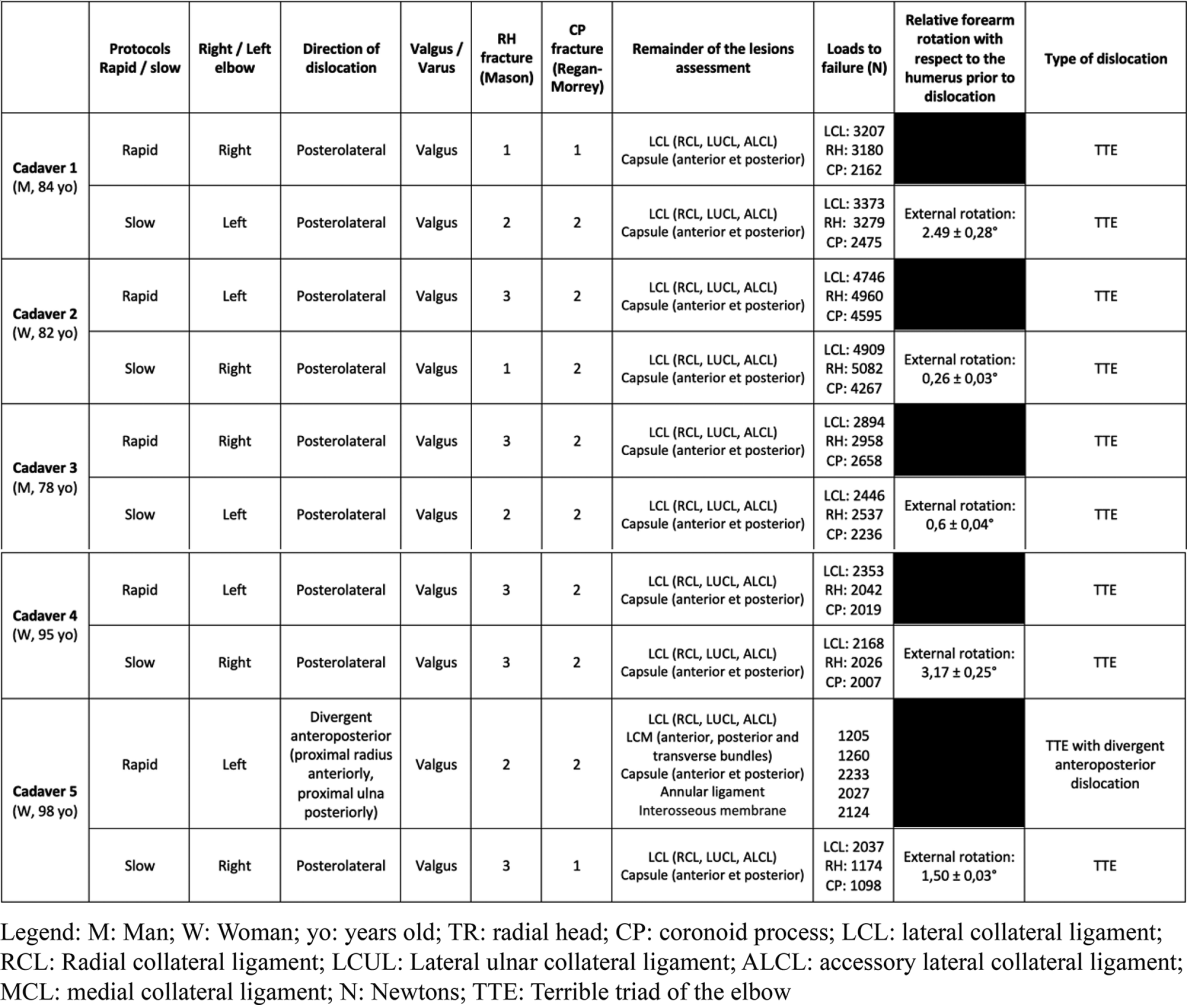
Lesion assessment, loads to failure and relative forearm rotation with respect to the humerus prior to dislocation

### Loads to failure

Table 1 shows loads to failure for each case.

Mean loads to failure were 3,126 ± 1,066 N for the LCL, 3,026 ± 1,308 N for the RH, and 2,613 ± 1,120 N for the CP.

**Fig. 4:**
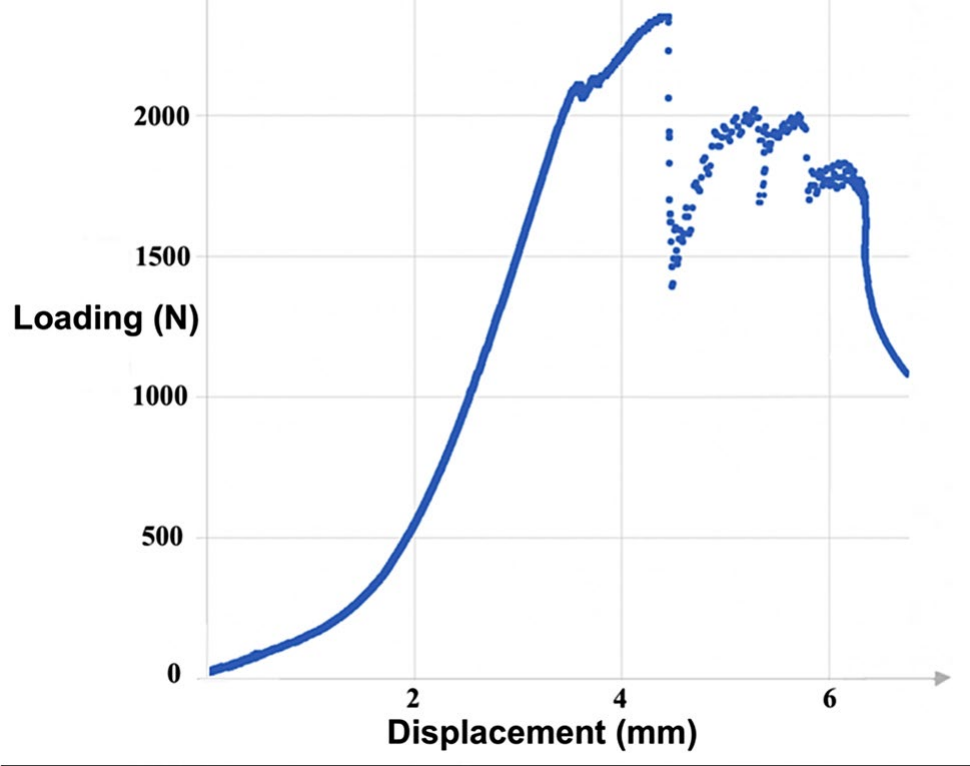
Typical load-displacement curve in posterolateral TTE (rapid protocol applicated on the left elbow of cadaver No. 4)

**Fig. 5:**
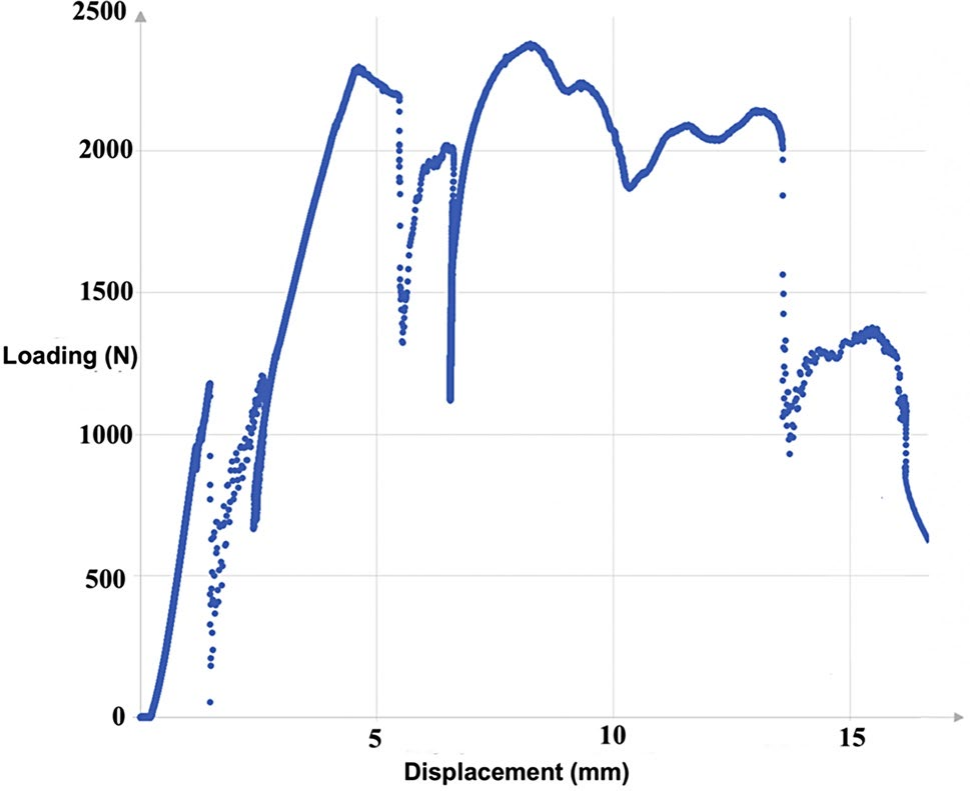
Typical load-displacement curve in divergent anteroposterior TTE (rapid protocol applicated on the left elbow of cadaver No.5)

Figures 4 and 5 respectively show load-displacement curves for the rapid protocol in cadaver 4 (TEE with posterolateral dislocation) and cadaver 5 (TTE with divergent anteroposterior dislocation).

Comparison between LCL, RH and CP loads to failure on the rapid protocol in cadavers 1 to 4 found a p-value of 0.39 for each.

Comparison of mean loads to failure for all structures in a given elbow on the rapid protocol in specimens 1 to 4 found a p-value of 0.03.

Comparison of the difference in LCL, RH and CP loads to failure between the slow and rapid protocols in cadavers 1 to 4 found a mean difference of -4%.

### Relative forearm rotation with respect to the humerus prior to dislocation

Table 1 shows relative forearm rotation with respect to the humerus prior to dislocation for each TTE.

The mean value was 1.6 ± 1.2°.

All rotations were external.

## Discussion

### Reproducibility of the anatomical model of TTE

These results allow us to answer our hypothesis. Indeed, among the 5 elbows of the two different protocols, ten TTE were created using the model: 9/10 (90%) with posterolateral and 1/10 (10%) with divergent anteroposterior dislocation.

The classic posterolateral dislocation in TTE has been reported previously [7, 8, 11]. Fitzpatrick & al. created 6 TTE with posterior dislocation under axial compression from 7 anatomical models positioned in pronation, but no randomization between models was performed [7]. Wake & al. created 11 TTE with posterior dislocation under axial compression from 15 elbows in full extension or 30° flexion; however, they did not detail pronation-supination and the models used formaldehyde-treated elbows, which would alter tissue properties and affect lesion onset [15].

Divergent dislocation was described radiographically by DeLee in 1981 [21], but is rarely seen in practice [22]. It includes 2 forms: anteroposterior divergence, which is the more common, and mediolateral divergence, less common [22–24].

In anteroposterior divergence, several studies have identified the mechanism as axial compression of the hand in extension, forearm in pronation and elbow in sub flexion, with a pivoting movement of the body relative to the hand. Thus, the lesion mechanism corresponded to the current test-bench position of the elbows, accounting for the creation of a single divergent anteroposterior TTE with probable error in fixation of the specimen on the test bench. Finally, the present study found 8 Regan-Morrey type-2 CP fractures and 2 type-1, in agreement with Doornberg & al., who reported that CP fracture in TTE occurred at approximatively 35% of CP height [25].

We reproduced TTE with the forearm in maximum pronation. This was chosen based on Fitzpatrick’s findings [7], with simple dislocation when the forearm was in maximal supination and associated RH fracture when in pronation. However, it is well described [10, 26] that the TTE lesion mechanism involves the elbow being in mild flexion and the forearm in supination. The present study casts doubt on this, as did Schreiber [17], whose video analysis found 70% of elbows in pronation at the time of trauma.

### Loads to failure according to model

To study loads to failure, we did not position sensors on the structures involved by TTE, as this would have required at least slight opening of the joint capsule, which would undoubtedly alter the biomechanical properties of the model.

During axial compression of the whole elbow resulting in TTE with posterolateral dislocation, there was systematic valgus displacement and pathological forced external rotation (PFER) of the forearm. This corresponds exactly to the description by O’Driscoll & al. [8, 10, 18]. Also, the lesion chronology in TTE with posterolateral dislocation has also been described in several studies and although other theories have been proposed [1, 17, 27–30], the main one is the Horii circle described by O’Driscoll & al. and adopted by many authors. This begins with capsule-ligament rupture, first in the LCL and progressively extending laterally and medially, with possible injury to the posterior and transverse MCL bundles and, less commonly, the anterior bundle. This is followed by RH and CP passage fractures during the posterior dislocation [5–10]. The present elbow displacement patterns corresponded exactly to this pathophysiology as described by O’Driscoll, with 3 loads to failure seen on the load-displacement curves and lesion assessment systematically finding LCL tear associated with RH and CP fractures, without visible tear in the MCL bundles. Capsule rupture is progressive under these circumstances [5–10] and was thus not seen as an inflexion in the load-displacement curve.

This precise chronology thus sequences failure loading in each structure of the triad: first, LCL tear, then RH fracture, and finally CP fracture.

Mean loads to failure, for all elbows of the 2 protocols combined, in the LCL were 3,126 ± 1,066 N, in the RH 3,026 ± 1,308 N and in the CP 2,613 ± 1,120 N.

To the best of our knowledge, Fitzpatrick & al.’s study was the only one to report a mean load to failure in TTE, at 2,355.4 ± 339.8 N, without, however, detailing the number of loads to failure observed or their association with damaged structures [7].

Amis & al. reported mean loads to failure of 2,900 N (range, 300-6,100) in the RH and 4,300 N (range, 1,600-6,000) in the CP; however, their study concerned fracture outside of TTE contexts, with a direct or indirect trauma protocol very different from that used in the present study [12].

Since dislocation is a sudden event [20], the rapid protocol more closely approximated in-vivo trauma.

In rapid protocols in cadavers 1 to 4, comparison of loads to failure per structure (LCL, RH, CP) found no significant differences (*p* = 0.39), with equal p-values for each structure, likely due to small numbers, whereas comparison of mean loads to failure in all structures in a given elbow found a significant difference (*p* = 0.03). This may be explained by differences in age (78–98 years) and gender between cadavers.

In the light of these differences between cadavers, we compared differences in loads to failure per structure (LCL, RH and CP) between the slow and rapid protocols in a given cadaver (1 to 4). This found a low mean value of -4%, with random homogeneous distribution around zero. In this small sample of 4 cadavers, this suggested that there was no systematic over- or under-estimation of loads to failure between the 2 protocols for a given cadaver.

In the present series there were no lesions in the anterior, transverse or posterior MCL bundles in TTE with posterolateral dislocation. Although several studies did report MCL lesions in simple or complex elbow dislocation [28, 30, 31], O’Driscoll did not and explained this classic absence of anterior MCL bundle lesion in TTE [32, 33] by the RH and CP fractures, which allowed much of the dislocation energy to dissipate [10].

### Relative forearm rotation with respect to the humerus prior to dislocation between models

Among the 5 elbows of the two different protocols, we found systematic external rotation of the forearm with respect to the humerus in all models under axial compression, with a mean 1.6 ± 1.2° prior to dislocation.

As described above, comparison of differential loads to failure per structure (LCL, RH, CP) between slow and rapid protocols in a given cadaver showed no systematic over- or under-estimation between the two. Thus, relative rotation values obtained with the slow protocol should be applicable to the rapid protocol.

Many authors have reported forearm PFER as the initial stage in posterior dislocation of the elbow [6, 11, 26]. It is defined as the combined relative rotation of the ulna with respect to the humeral trochlea and of the radius with respect to the capitulum, as distinct from pronation-supination defined as the relative rotation of the radius with respect to the ulna [7]. It is explained by the inclined surface of the lateral part of the medial two-thirds of the humeral trochlea, converting forearm axial compression force into lateral rotation [27].

However, PFER, does not appear to be systematic: Fitzpatrick & al. reported that 4 out of the 6 TTE they created started with PFER with initial LCL tear, and 2 with forced forearm internal rotation with initial MCL tear, and concluded that whether forearm rotation was lateral or medial determined whether the LCL or the MCL was torn first [7].

### Study limitations and strengths

To our knowledge, this was the first study to create a reproducible anatomical model of TTE, and measure relative forearm rotation with respect to the humerus prior to dislocation and loads to failure in each structure involved by TTE. Previous studies focused on the theoretical, pathophysiological or therapeutic principles of TTE [8, 10, 16, 17, 28, 29], or on the biomechanical consequences of each individual lesion [5, 6, 13, 14], sometimes including anatomic models of elbow dislocation, but none have addressed the specific issues of TTE, and moreover all had serious limitations [7, 12, 15]. Another strength was the use of fresh specimens, without the formaldehyde used in other studies [15] which is known to alter the biomechanics of tissue by solidifying and increasing stiffness [34, 35]. Other strengths were that randomized matching between the slow and rapid protocols avoided numerous confounding factors, and the study was conducted in a laboratory certified and specializing in biomechanics. Finally, reproducibility between models was ensured by a precise dissection technique performed by a single surgeon, with standardized positioning on the test bench in constant 15° flexion and maximal pronation.

Nevertheless, 1 TTE showed divergent dislocation while the contralateral elbow from the same cadaver and the other 8 models showed posterolateral dislocation, suggesting that reproducibility could be further improved, as a lack of reproducibility in flexion may affect the lesion that is created. Wake & al. showed that, when elbow flexion increased under axial compression, the compression forces shifted from the CP toward the olecranon [15].

The other study limitations were the lack of bone density quantification by imaging which is inherent to any in-vitro study, making extrapolation to clinical settings.

## Conclusions

The present results demonstrated that we were able to create and validate an anatomical model of TTE by exerting axial compression on an elbow in 15° flexion and maximal pronation at speeds of 100 and 10 mm/min.

## Data Availability

No datasets were generated or analysed during the current study.

## Abbreviations

CP: Coronoid Process
Hz: Hertz
LCL: Lateral Collateral Ligament
MCL: Medial Collateral Ligament
Mm: Millimeters
N: Newtons
PFER: Pathological Forced External Rotation
RH: Radial Head
TTE: Terrible Triad of the Elbow
